# Decoding uncertainty for clinical decision-making

**DOI:** 10.1098/rsta.2024.0207

**Published:** 2025-03-13

**Authors:** Krasimira Tsaneva-Atanasova, Giulia Pederzanil, Marianna Laviola

**Affiliations:** ^1^ Department of Mathematics and Living Systems Institute, University of Exeter, Exeter, UK; ^2^ Computational Science Laboratory, Informatics Institute, University of Amsterdam, Amsterdam, UK; ^3^ Injury, Recovery and Inflammation Sciences Academic Unit, School of Medicine and National Institute for Health and Care Research, Nottingham Biomedical Research Centre, University of Nottingham, Nottingham, UK

**Keywords:** uncertainty quantification, medical innovation, evidence-based medicine, clinical data analysis, diagnostic tools, precision medicine

## Abstract

In this opinion piece, we examine the pivotal role that uncertainty quantification (UQ) plays in informing clinical decision-making processes. We explore challenges associated with healthcare data and the potential barriers to the widespread adoption of UQ methodologies. In doing so, we highlight how these techniques can improve the precision and reliability of medical evaluations. We delve into the crucial role of understanding and managing the uncertainties present in clinical data (such as measurement error), diagnostic tools and treatment outcomes. We discuss how such uncertainties can impact decision-making in healthcare and emphasize the importance of systematically analysing them. Our goal is to demonstrate how effectively addressing and decoding uncertainties can significantly enhance the accuracy and robustness of clinical decisions, ultimately leading to better patient outcomes and more informed healthcare practices.

This article is part of the theme issue ‘Uncertainty quantification for healthcare and biological systems (Part 1)’.

## 1. Introduction

In healthcare, clinical decision-making is a critical process that directly affects patient outcomes. However, the inherent uncertainties in medical data, patient responses and treatment outcomes pose significant challenges. These uncertainties can stem from various sources, including variability in patient characteristics, limitations of diagnostic tests and the complex nature of diseases. As healthcare professionals strive to provide the best possible care, understanding and managing these uncertainties is crucial for improving the accuracy and effectiveness of clinical decisions.

Uncertainty quantification (UQ) is a scientific discipline focused on the systematic analysis and management of uncertainties in mathematical and statistical models as well as data-driven simulations [1]. It provides a structured framework for understanding how variability and errors in model inputs propagate to affect the outputs. Indeed, there can be measurement errors in model inputs (e.g. the precision of the measurement of blood pressure) or even natural variability (e.g. one’s blood pressure is different on different days), and it is crucial to take into account how this affects the model results.

The inclusion of computational modelling and simulation in clinical practice has the potential to significantly improve patient care, but so far, the rate of adoption of such models has been extremely limited compared with the maturity of the field. Although the reasons for this are many [2], one aspect is certainly the burden on the modelling community to demonstrate model *credibility*, i.e. provide extensive evidence that the model is trustworthy and effective in improving patient care. The three pillars of model credibility are *verification*, *validation* and *uncertainty quantification* [3]. Verification is the analysis that the computational implementation of the mathematical model is correct, i.e. that the computer code solves the model equations correctly. Validation is the test that the model equations capture the behaviour of interest of the system under consideration, in other words, that model results match related experimental measurements. Finally, as mentioned earlier, UQ is the study of how uncertainties in model inputs affect model outputs. While verification is crucial for ensuring a model’s reliability, often to the point of being taken for granted and validation provides the most direct proof of a model’s utility for its intended purpose, uncertainty quantification is an equally important aspect that is perhaps currently underestimated.

**Figure 1.**
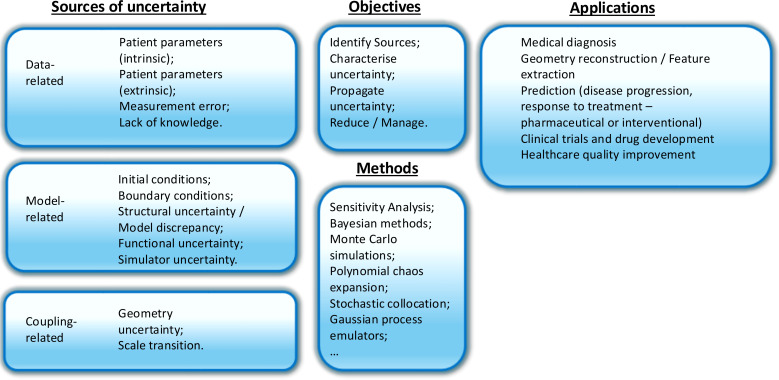
Key insights and structure of the opinion piece.

In this opinion piece, we aim to highlight the role of UQ in clinical decision-making and its potential to enhance the quality of patient care and transform clinical practice. The manuscript is organized as follows: in §2, we list possible sources of uncertainty in models related to healthcare; in §3, we define UQ goals and methodologies that are specific to the context of healthcare; in §4, we discuss applications of UQ in healthcare, such as personalized and optimized patient treatment, enhanced diagnostic accuracy, improved risk management and robust clinical trials and drug development; and finally, in §5, we discuss current challenges to the adoption of UQ in healthcare and recommendations for improvement (see also figure 1).

## 2. Sources of uncertainty in healthcare

In the context of healthcare, UQ means understanding how uncertainties in patient data and (bio)medical models affect the outcomes of clinical decisions. In this section, we list and discuss possible sources of uncertainty for healthcare-related models, but before we do that it is useful to clarify some details about the kinds of models we are considering. Models can be generally considered either *knowledge-driven*, *data-driven* or *hybrids* of the former two.

— *Knowledge-driven* models are based on laws that characterize the system we are looking to model, often in the form of equations describing either the transformation of an input into an output or the evolution of the system over time. Often they are based on the laws of physics, chemistry and/or biology and involve solving boundary value problems, i.e. ordinary or partial differential equations paired with appropriate initial conditions, boundary conditions and a geometry of interest. Examples of these are pharmacokinetic models, electromechanical models of the heart or computational fluid dynamics simulations of blood flow in a circulatory system. Other kinds of knowledge-driven models are system or network dynamics models, for example, models of resource use in a hospital or of the transport logistics of a medical drug.
— *Data-driven* models make no assumptions about the laws behind the system of interest but are purely data analysis tools that aim to extract information from (usually large) datasets. Examples of these are machine learning or deep learning models, as well as statistical models such as Bayesian network analysis.
— *Hybrid* models are, as the name suggests, models that combine both approaches. Examples of these are physics-based neural networks or medical image processing algorithms that use both deep learning methods as well as knowledge of the physics of the relevant imaging systems.

We would like to highlight, in particular, some model types that require special consideration. One example is *medical image processing* algorithms since, depending on the use case, they can be considered as a self-contained model or as one step in a larger simulation. For example, an algorithm that quantifies the amount of extravasated blood in a cerebral haemorrhage patient when the clinical decision is based solely on the number that quantifies that volume is a model on its own. On the other hand, an algorithm that reconstructs a geometry, which is then input into a boundary value problem as the domain of interest, is part of a larger simulation. The relevance of this part of the simulation was highlighted, for example, in [4]. Another example is given by *clinical outcome mapping* algorithms: these are used to transform the outcome of a knowledge-driven model, which is often a physics-/chemistry-/engineering-based quantity such as a stress distribution or a density distribution of a chemical of interest, into a clinical outcome such as pain, hospitalization, morbidity score or mortality.

Sources of uncertainty are several and other authors have suggested lists in the context of healthcare, among others [5,6]. We propose a (likely not exhaustive) list in the following, where we group the possible sources for convenience as *data-related* (a.k.a. aleatoric), *model-related* (a.k.a. epistemic) or *coupling-related*, where the latter refers to parts of models that are self-contained algorithms and that for the purposes of verification, validation and uncertainty quantification (VVUQ) activities can be treated either as a model of their own or as a component of a larger model.

### (a) Data-related uncertainties

— *Intrinsic variability of model inputs* is the time-dependent variation of model input, for example, a patient’s blood pressure reading changing throughout the day or on different days.
— *Extrinsic variability of model inputs* is the sample-dependent variation of a model input, such as patient-specific variability of genetics, physiology and lifestyle.
— *Measurement error* is due to the finite precision of measuring instruments, for example, a patient’s weight depending on the precision of the scale.
— *Lack of knowledge* is due to incomplete or missing data, such as incomplete medical records, data entry errors or missing fields in record templates that are relevant to advanced knowledge; it can also be due to patient non-compliance, fragmented healthcare records or insufficient medical history documentation [7,8]; finally, it can relate to the lack of consideration (often because it would be an overwhelmingly complex thing to do) of the interactions between different conditions, such as the case of a patient with two or more conditions simultaneously (e.g. diabetes and cardiovascular disease), where the two are addressed separately.

For example, a source of uncertainty stems from the limitations of diagnostic tests, as no diagnostic test is perfect. Tests can yield false positives or false negatives and their accuracy can be influenced by factors such as the stage of the disease, the presence of other medical conditions and technical limitations.

Imaging tests such as MRI or CT scans can miss small tumours or lesions, leading to uncertainty in diagnosing conditions such as cancer [9,10]. Often, clinicians must make decisions based on incomplete medical histories or limited patient data. This lack of comprehensive information can lead to uncertainty in diagnosing conditions or choosing the best course of treatment. Many diseases, particularly chronic and complex ones, have multifactorial causes and diverse presentations. Conditions such as diabetes, heart disease and autoimmune disorders involve intricate interactions between genetic, environmental and lifestyle factors. This complexity adds layers of uncertainty to predicting disease progression and treatment responses [11,12].

### (b) Model-related uncertainty

— *Initial and boundary conditions* are relevant when the model is a boundary-value problem for which not only the differential equations but also initial and boundary conditions must be supplied (e.g. the microvascular resistance downstream of a coronary artery).
— *Model discrepancy* encompasses all sources of mismatch between a model and reality, while structural uncertainty specifically addresses uncertainty about a model’s assumptions. In that sense, structural uncertainty contributes to model discrepancy. One example of structural uncertainty in healthcare applications is the lack of consideration of, for example, the genetics of a disease (perhaps for lack of knowledge), which is therefore entirely omitted from the model; another example is the isolation of a disease from other conditions when a patient might be affected by several conditions at once (e.g. diabetes and cardiovascular disease).
— *Functional* uncertainty refers to the error bounds on the range of applicability of a given model, such as a model being valid for patients within a given age range.
— *Simulator* arises from the necessary discretizations and numerical approximations made during the computational implementation.
— *Output* is the final uncertainty accompanying the output of the model.

### (c) Coupling-related uncertainty

— *Geometry* uncertainty relates to any model that requires estimating geometrical features related to a patient’s condition, for example, segmentation and reconstruction of patient-specific organs, blood vessels or similar feature extraction; these can be either the endpoint of a model (e.g. estimation of blood volume in a heart chamber) or the domain of a boundary value problem.
— *Scale transition* uncertainty pertains to multiscale models where lower-level dynamics (e.g. diffusion of signalling molecules within a cell) have to be translated into one ‘global’ parameter at the higher level (e.g. chemical concentration in a tissue volume element); this can also be considered a specific type of model discrepancy.

Although not all sources of uncertainty will be relevant for all models, any model is likely to deal with several of them, and they will also interact in complex ways. By employing advanced statistical and computational techniques, UQ provides a framework for quantifying and managing these uncertainties. This could not only enhance the reliability of clinical decisions but also support the development of personalized treatment plans tailored to individual patients’ needs. Incorporating UQ into models can improve their effectiveness and foster greater trust in their predictions. These differences can significantly impact how patients respond to treatments and interventions. For instance, a medication that works well for one patient may be less effective or cause adverse effects in another due to genetic differences. Even with well-established treatments, predicting outcomes can be challenging. Factors such as patient adherence to treatment regimens, individual variations in drug metabolism and the presence of co-morbid conditions can affect how a patient responds to therapy. For instance, the effectiveness of chemotherapy can vary widely among cancer patients due to genetic differences in drug metabolism and tumour biology [13,14].

## 3. Defining UQ in the context of healthcare

In the context of healthcare, UQ helps quantify how uncertain factors such as patient data variability, diagnostic inaccuracies and treatment responses impact clinical decisions and patient outcomes. The primary goals of UQ are to:

— *Identify sources of uncertainty*—Determine the origins of uncertainties within the model, whether they stem from data inaccuracies, model assumptions or external factors.
— *Characterize uncertainty*—Use statistical techniques to describe the uncertainties quantitatively, often through probability distributions, confidence intervals and other metrics.
— *Propagate uncertainty*—Analyse how uncertainties in inputs affect the outputs of the model, allowing for a comprehensive understanding of the potential variability in outcomes.
— *Reduce and manage uncertainty*—Implement strategies to mitigate the impact of uncertainties on decision-making, thereby enhancing the reliability and robustness of clinical predictions and interventions.

We outline below several methodologies commonly employed in UQ to achieve these goals:

— *Probabilistic methods*—Probabilistic models use probability distributions to represent uncertainty in model inputs and outputs [15]. These models can capture the inherent randomness and variability in clinical data. For instance, a probabilistic model might represent the distribution of possible blood pressure readings for a patient population, accounting for measurement errors and biological variability. Examples include but are not limited to:
  - *Bayesian methods*—Bayesian methods incorporate prior knowledge along with new data to update the probability estimates of uncertain parameters [16]. This approach is particularly useful in clinical settings where prior studies or expert knowledge can inform the analysis. For example, Bayesian methods can update the probability of a disease diagnosis as new diagnostic test results become available.
  - *Monte Carlo simulations*—Monte Carlo simulations use repeated random sampling to explore the range of possible outcomes given uncertain inputs [17]. This technique can generate a distribution of potential outcomes, providing insights into the likelihood of different scenarios. In clinical practice, Monte Carlo simulations might be used to predict the range of potential outcomes for a new treatment, considering patient variability and uncertainty in treatment response.
— *Sensitivity analysis*—Sensitivity analysis examines how changes in model inputs affect outputs [18,19]. By identifying which inputs have the most significant impact on outputs, sensitivity analysis helps prioritize areas for reducing uncertainty. For example, sensitivity analysis can determine which patient characteristics (e.g. age, co-morbidities) most influence the effectiveness of a treatment.
— *Uncertainty propagation techniques*—These techniques involve mathematical methods to propagate input uncertainties through the model to the outputs. Methods such as polynomial chaos expansion [20,21] and stochastic collocation [22] are used to efficiently compute the impact of uncertainties on model predictions.

The above list is not exhaustive but provides a representative selection of widely used techniques in uncertainty quantification, highlighting both probabilistic and non-probabilistic approaches. Each method has unique strengths and applications, and in practice, multiple techniques are often combined to achieve a more comprehensive assessment of uncertainty in complex models such as those arising in the context of healthcare.

## 4. Applications of UQ in healthcare

Computational models incorporating UQ can be used at different points along the healthcare pathway and clinical decision-making process, depending on the specific application and the type of uncertainty being considered. Clinical decision support (CDS) systems commonly employ computational models and technologies to assist clinicians in making decisions about patient care. CDS systems have the potential to improve patient outcomes and reduce healthcare costs by providing clinicians with real-time, evidence-based recommendations for patient care. However, the translation and application of CDS systems in clinical practice can be complex and challenging, and UQ has an important role to play. In the context of CDS and UQ, it is essential to use high-quality data from well-designed clinical studies that are relevant to the specific clinical question being addressed to ensure safe decision-making. The data used should also be representative of the population of interest, as differences in patient characteristics can impact the accuracy and reliability of the models. Data evaluation and data harmonization play a big role in this context because data evaluation ensures that they are suitable for use in the model and considers appropriate UQ methodology and data harmonization techniques ensure consistency and compatibility through the process of standardizing data from different sources.

Uncertainty can lead to diagnostic errors, including misdiagnoses or delayed diagnoses. Choosing the most effective treatment often involves navigating through uncertain outcomes. Clinicians must weigh the potential benefits and risks of various options, sometimes without clear evidence favouring one approach over another. Uncertainty can compromise patient safety if it leads to inappropriate treatment choices or missed diagnoses. For instance, prescribing a medication without fully understanding its potential interactions with other drugs the patient is taking can lead to adverse effects. Uncertainty can drive up healthcare costs due to the need for additional tests, treatments and hospitalizations resulting from diagnostic errors or ineffective treatments.

To effectively translate and apply CDS systems in clinical practice, the following considerations should be taken into account:

— *User-centred design*—CDS systems should be designed with input from end-users, including clinicians and patients, to ensure that they are user-friendly and aligned with the needs of the intended users.
— *Interoperability*—CDS systems should be interoperable with existing electronic health record (EHR) systems and other clinical workflows to facilitate seamless integration and use.
— *Data quality and standardization*—High-quality data that is standardized across multiple sources is essential for accurate and reliable CDS system recommendations. Efforts should be made to ensure that data is accurate, complete and consistent.
— *Clinical guidelines and evidence-based* *practices*—CDS systems should be based on clinical guidelines and evidence-based practices to ensure that recommendations are aligned with current best practices.
— *Evaluation and validation*—CDS systems should be evaluated and validated in real-world settings to ensure that they are accurate, effective and have a positive impact on patient outcomes.
— *Privacy and* *security***—**Patient privacy and data security should be a top priority in the development and deployment of CDS systems. Appropriate security measures should be implemented to protect patient data and prevent unauthorized access.
— *Education and training***—**Education and training are essential to ensure that clinicians and the next generation of medical doctors understand how to use CDS systems effectively and integrate recommendations into their clinical decision-making processes.

Uncertainty is an unavoidable aspect of clinical decision-making, but by understanding its sources and impacts, healthcare professionals can adopt strategies to mitigate its effects. Embracing UQ and evidence-based practices can lead to more informed and effective clinical decisions, ultimately improving patient outcomes and the overall quality of care.

Incorporating UQ into clinical decision-making is crucial for *diagnostic accuracy* as it enhances the accuracy of diagnostic tools by quantifying the uncertainty in test results. For example, in medical imaging, UQ methods can assess confidence in detecting abnormalities, thereby aiding radiologists in making more accurate diagnoses [23]. UQ enhances diagnostic accuracy in medical imaging by quantifying uncertainties in image interpretation and detection algorithms [24,25]. For example, in radiology, UQ can be used to assess the confidence levels in detecting tumours or other abnormalities in MRI or CT scans [26]. This helps radiologists distinguish between true positives and false positives, thereby reducing diagnostic errors. In medical image synthesis, UQ methods can enhance clinicians’ trust in machine-learning solutions by explicitly addressing and managing the uncertainties involved [27]. In biomarker analysis, UQ enables quantification of the variability and reliability of biomarker levels used for disease diagnosis. For instance, in diagnosing conditions like prostate cancer using prostate-specific antigen levels, UQ can account for measurement errors and biological variability, potentially leading to more accurate and reliable diagnostic decisions [28,29]. A recent review [5] found that in the last decade, studies focused on medical images, with a prevalent application of UQ techniques using deep learning models compared with machine learning models and a few studies applied UQ to physiological signals. UQ techniques using machine learning models have primarily been applied to neurological systems, followed by thoracic (cardiac) systems, medical data, other organs (breast cancer detection is the most studied), musculoskeletal and digestive systems. On the other hand, UQ techniques using deep learning models focused on brain, eye and skin images, followed by chest, cardiac and breast images.

UQ is essential for *risk assessment* in surgeries and other medical procedures. By analysing uncertainties in patient health status, UQ supports the identification and mitigation of potential risks, improving patient safety. For example, the need for methods that quantify model uncertainty in diagnostics arises from the invasive, costly and time-consuming nature of angiography, the gold standard for diagnosing coronary artery disease, a leading global cause of death. Non-invasive computational algorithms addressing this uncertainty have demonstrated high diagnostic accuracy for identifying stenosis in major coronary arteries [30]. Moreover, based on patient-specific data and surgical factors, UQ can predict potential complications and outcomes. For instance, in cardiac surgery, UQ models can estimate the risk of postoperative complications based on patient health metrics and surgical variables, aiding surgeons in preoperative planning and decision-making [31,32].

Employment of computational models incorporating UQ in the development of *personalized treatment plans* is also a very promising application of UQ in healthcare. Such models can be used to predict the likely outcomes of different treatment options for individual patients, taking into account the uncertainties associated with patient physiology, medical test results and other relevant factors. This can help clinicians make more informed treatment decisions and improve patient outcomes. For example, UQ plays a critical role in oncology by improving the precision of treatment plans. Indeed, UQ can help in developing personalized treatment plans by evaluating the uncertainties in tumour growth models and treatment response predictions. This ensures that treatment strategies are tailored to individual patient profiles, maximizing efficacy and minimizing adverse effects [33]. In radiation therapy, UQ is used to model the uncertainties in tumour positioning and patient movement, ensuring that radiation doses are accurately targeted to maximize efficacy while minimizing damage to healthy tissues [34–36]. In pharmacotherapy, UQ helps personalize medication regimens by accounting for uncertainties in drug metabolism and patient response. For instance, in treating chronic diseases like diabetes, UQ models can predict how different patients will respond to insulin therapy, leading to tailored treatment plans that optimize blood sugar control [37–39].

Another potential application is in *clinical trials and drug development*. UQ can be used to model the variability in patient responses to a particular drug, for example, or to estimate the probability of success for a new treatment based on clinical trial data [40]. In clinical research, UQ helps in the design and interpretation of trials by quantifying the uncertainties in experimental conditions and measurement processes. This ensures that clinical trials are statistically sound and that their results are reliable and reproducible. For example, UQ can guide sample size determination and experimental protocols in drug efficacy studies [40–42]. UQ is crucial for interpreting clinical trial results, as it quantifies the uncertainty in outcome measures and treatment effects. This helps researchers and clinicians understand the range of possible effects of treatment and the likelihood of different outcomes, leading to more informed clinical guidelines and practices [40,43]. Accurate forecasting of clinical trial approvals is essential to allocate resources efficiently, but existing prediction algorithms lack uncertainty quantification and interpretability, limiting their practical application in clinical trial management [44].

In addition, UQ can be used in *healthcare quality improvement* efforts. Large-scale simulation models are being increasingly used for evaluating the cost-effectiveness of medical interventions. Given their intricate nature and the large number of parameters they incorporate, traditional methods for quantifying parameter uncertainties, such as Monte Carlo sampling, can be excessively costly. To address this challenge, Zheng & Dinh [45] developed a robust and efficient methodology for quantifying parameter uncertainties in such large-scale simulation models. Specifically, to enhance traditional probabilistic sensitivity analysis, Zheng & Dinh [45] take a four-step approach: (i) surveying all parameters and their confidence intervals, (ii) performing local sensitivity analysis to assess each parameter’s effect, (iii) ranking key parameters with the highest impact and (iv) building response surface approximations using Latin Hypercube sampling and multivariate adaptive regression splines to model outcomes. Moreover, by quantifying the uncertainties associated with different healthcare processes and interventions, healthcare providers can identify areas for improvement and develop more effective quality improvement strategies. UQ informs health policy decisions by providing a robust framework for evaluating the uncertainties in public health models and forecasts [46]. For instance, in planning vaccination campaigns, UQ can model the uncertainties in vaccine efficacy and population immunity, helping policymakers design effective strategies to control disease outbreaks [47–49]. In epidemiology, UQ is used to model the spread of infectious diseases and assess the uncertainty in epidemiological forecasts. During the COVID-19 pandemic, UQ models helped predict infection rates and assess the impact of public health interventions, providing crucial insights for policymakers and healthcare providers [50–53]. UQ aids in optimizing resource allocation in healthcare systems by identifying areas with the highest uncertainty and potential impact. This ensures that resources such as medical staff, equipment and funding are directed to areas where they are most needed and can be most effective [46,54].

## 5. Challenges and recommendations

Implementing UQ in clinical settings presents several challenges, including ensuring high-quality data, managing computational complexity, tackling computational costs and fostering interdisciplinary collaboration. Data quality is crucial, as unreliable or incomplete data can lead to inaccurate UQ results. Priority should be given to improving data quality (i.e. evaluation of factors such as sample size, patient characteristics and data completeness) and a better understanding of the computational complexity that arises from uncertainties from different sources (such as patient variability, diagnostic test limitations, limited or missing data and model discrepancy). Importantly, the computational demands of UQ methods can be significant, requiring advanced tools and resources that may not be readily available in all clinical environments. The implementation of UQ would require thousands of simulator evaluations, with significant costs and complexity making such implementations unfeasible in the clinical routine. In such cases, UQ methodology may use a surrogate model, a computationally inexpensive approximation of the simulator built from a limited number of simulator evaluations, which can be classified into two categories: projection-based reduced-order model and data-fit model [55]. This may be a choice to have predictive accuracy with a significant reduction of computational cost [56].

Moreover, effective UQ often necessitates collaboration across various fields—such as data science, clinical practice and statistics—requiring seamless communication and integration of expertise to successfully apply these techniques in clinical settings. As scientists, modellers and mathematicians, we should collaborate with clinicians, medical doctors and healthcare professionals to address the major challenges posed by the implementation of UQ in clinical settings.

The path to adoption of biomedical software into clinical practice can be thought of as having two stages: one is the obtaining of regulatory approval and the other is the actual adoption in clinical practice, where the two potentially overlap partially if a human clinical trial is required. The first stage is challenging due to the lack of harmonized regulatory requirements between different agencies, such as the Food and Drug Administration (FDA) and the European Medicines Agency and the complexity of determining what extent of credibility activities is necessary and sufficient. The modelling community has been discussing guidelines within itself and has been sharing experiences, though some challenges remain, such as the strong dependence of VVUQ activities on specific characteristics of each model (making universal guidelines challenging and the sharing of individual experiences only partially useful), the potentially limited access to resources for experimental validation (benchtop equipment, animal studies or human clinical trials) and the current lack of universal standards for biomedical models [57–59].

Future research and development in UQ should focus on several key areas to enhance its application in clinical settings. First, there is a need for advanced algorithms that can efficiently handle the complexities of clinical data and provide more precise uncertainty estimates. These algorithms should be designed to work with large, heterogeneous datasets and improve computational efficiency. Second, better data-integration strategies are essential to unify disparate data sources, ensuring that UQ methods have access to comprehensive and high-quality information. This will involve developing frameworks for integrating EHRs, imaging data and other relevant clinical data. Last but not least, training for healthcare professionals is crucial to bridge the gap between sophisticated UQ techniques and practical clinical use. Educational programmes and resources should focus on equipping clinicians with the skills to interpret UQ results and apply them effectively in decision-making processes. By addressing these areas, we can advance UQ methodologies and improve their utility and impact in clinical practice.

Enhanced collaboration between modellers, statisticians and healthcare professionals is essential. While awareness of the importance of UQ is growing within the modelling community, its integration into routine model development remains limited. Modellers may benefit from exposure to new techniques and advancements that they may not be aware of. Statisticians, on the other hand, need to understand the complexities of modelling pipelines to guide modellers effectively in selecting the most appropriate UQ analyses. For healthcare professionals, it is crucial to establish confidence in the mathematical validity of UQ analyses to trust model predictions and understand their limitations. Clinicians also emphasize that early involvement in the modelling process enhances adoption, ensuring that the model is tailored to address the specific clinical decisions they face. Therefore, fostering this interaction is highly desirable to align model development with clinical needs.

There is growing momentum to emphasize the importance of uncertainty quantification. Although one might think that validation is the most crucial step in this process, the authors believe uncertainty quantification will play a crucial role in establishing the trustworthiness of a model, especially when it is tasked with capturing a wide range of patient characteristics, which is the promise of *in silico* medicine. Although UQ plays a key role, modellers are often not familiar with what tools are available to them and/or which are more appropriate for their specific modelling framework. There is an opportunity here to either improve the modelling community’s knowledge of UQ methods, for example, by organizing workshops or curating freely available online material, or to recommend increased collaboration with experts in statistics and UQ methods to maximize the efficiency and effectiveness of model development. For both modellers and statistical experts, there is a challenge in the lack of consolidated regulatory pathways to guide the process and define the level of analysis necessary and sufficient for model adoption, as indicated in [2] among the barriers to clinical adoption. In the United States, recommendations from the FDA closely follow the ASME VV40 (2018) [3], a set of guidelines on verification and validation (VV) written by the American Society of Mechanical Engineers, with volume 40 being dedicated to *in silico* medicine. Moreover, some guideline papers have been published to further help modellers in planning appropriate VVUQ activities for their models, with a special focus on *in silico* clinical trials [60,61]. In the EU, IEC and ISO are working on a standard similar to the FDA’s, ISO/TS 9491, ‘Predictive computational models in personalized medicine research’: Part 1 was recently published and is concerned with ‘Constructing, verifying and validating models’ [62], while Part 2, ‘Guidelines for implementing computational models in clinical integrated decision support systems’, is currently under development.

It is worth reminding that a model VVUQ [63,64] activity must be contextualized to its predefined *context of use* (CoU) [3]. Indeed, regulatory approval of a model can only come when it is paired with a specific CoU, i.e. the definition of the bounds within which the model is valid and applicable as well as its role in informing the clinical decision (for example, will it be the only source of information in the clinical decision or will other data be used?).

The extensiveness of the required VVUQ activities is then dependent on the *model risk*, which is classified as low, medium or high according to a combination of *model influence* (how directly the model results affect the clinical decision) and *decision consequence* (the severity of the impact of an incorrect clinical decision on the patient’s health). Indeed, another important barrier to the application of UQ analysis is the computational cost, which can be very high when many simulations are required. Sometimes access to high-performance computing facilities is necessary to conduct UQ computations within a reasonable time, and this is not always possible. This limitation is acknowledged, for example, by the FDA and two considerations are possible: (i) for low-risk models, a smaller UQ campaign can be sufficient or (ii) reduced-order models or surrogate models are used, although this can require further validation that they can indeed replace the original model with sufficient accuracy.

## 6. Conclusion

Overall, UQ is a powerful tool that brings rigour and clarity to the inherently uncertain world of clinical decision-making. By embracing UQ, healthcare providers can navigate the complexities of medical practice with greater confidence, ensuring that patient care is both scientifically grounded and responsive to individual needs. The adoption of UQ in clinical decision-making offers numerous benefits.

— *Improved reliability*—UQ could enhance the reliability of clinical models and predictions by bringing in a systematic analysis of uncertainties.
— *Enhanced patient outcomes*—better understanding and management of uncertainties lead to more accurate diagnoses, effective treatments and personalized care plans, ultimately improving patient outcomes.
— *Informed decision making*—UQ could provide healthcare professionals with a clearer picture of the potential risks and benefits of different interventions, leading to better risk management, informed health policy decisions and hence more robust and confident decision-making.
— *Resource optimization*—UQ could help prioritize resource allocation and focus efforts on reducing critical uncertainties by identifying key areas of uncertainty, thus leading to more reliable, efficient and effective healthcare delivery.

Therefore, incorporating UQ in healthcare models empowers clinicians to make more informed, reliable decisions, ultimately enhancing patient outcomes and strengthening the healthcare system.

## Data Availability

This article has no additional data.
